# The Growth of the Nursing Profession: V. Knowledge and Practice in Nursing

**Published:** 1918-03-16

**Authors:** 


### THE MATRONS' AND SISTERS' DEPARTMENT.

### THE GROWTH OF THE NURSING PROFESSION.

### V. Knowledge and Practice in Nursing.

SOME of the educational problems presented by the actual training of nurses have already been dealt with in a series of articles appearing in this paper, and their importance cannot be too frequently emphasised. Our hospitals exist primarily for the benefit of the sick poor, that they may receive the surgical and medical treatment and the nursing care which for them would otherwise be inaccessible. Intimately connected with this primary object—so intimately that it cannot be regarded as a secondary one—is the training of those who are to give this surgical and medical treatment and nursing care, not only in the hospitals, but also in the homes of every class of the community affected by sickness. The nursing care involves the training of nurses, and although it would be impossible in the limits of a short article to go into even one of the complicated questions that are bound up with this matter, we might turn our attention for a few moments to the aim we have in view in considering the question at all. It may be said that it is waste of time making paper schemes which will be rendered useless by the practical difficulties which they are sure to encounter at every turn. True, but at the same time, if the College at the outset hitches its wagon to a star, it will in the end rise higher.

Why do we want to train nurses at all? What do we expect them to know, and why must they know it? Foolish questions perhaps, but elementary truths are sometimes forgotten in the mass of material with which they are overlaid. We train nurses because to ordinary men and women sickness is an unknown quantity which they do not understand. They are ignorant of its cause, of its possible results, and of the means of alleviating it. Ignorance means fear, fear means clumsiness and stupidity, and inability to do the right thing at the right time. Fear and clumsiness and stupidity all react upon the patient and retard his recovery. For this reason we train nurses and give them such knowledge as will eliminate fear, clumsiness, and stupidity, and create the best atmosphere for the work of the surgeon and the physician and of Nature herself.

What does the requisite knowledge include? The British nursing world as a whole has a great admiration for the American nursing world. The thoroughness of their organisation puts us to shame, they have a free and independent spirit, their nurses take a keen interest in the welfare and development of their profession, and American nursing text-books are to be found in every English nursing library. Take an average American nurse and an average English nurse of equal professional standing. Examine them in the theory of nursing or let them talk about the theoretical side of their profession, and the American will in all probability score at every point, the Englishwoman either having to bluff or to confess that she is out of her depth. But put those same two women to nurse two identical cases—difficult cases involving constant and intelligent care and attention, and we venture to say that the condition of the English nurse's patient will compare favourably with that of the patient nursed by the American. Why? Because in America so much attention is paid to theory that the actual practical details of nursing are not sufficiently emphasised.

The foregoing is, of course, a generalisation, and generalisations should always be handled carefully. But at least it can serve as a useful warning. More and more stress is being laid in recent years on the need for lectures, text-books, study, and accurate knowledge, and it is very right that this should be so; every increase of knowledge brings to the nurse increase of interest and of confidence. But in nursing, as in many other sciences, it has been proved that unless theoretical knowledge is accompanied by a proportionate ability to apply that knowledge the result will be disastrous.

We hear a great deal about the unnecessary amount of menial work imposed on nurses during their training, and undoubtedly reform in that direction is urgent. But it must be remembered that the ordinary comfort and well-being of the patient are every bit as important as the special treatment of his particular disease, and that the nurse is primarily responsible for these. We give the nurse knowledge in order that fear and clumsiness may be eliminated from the surroundings of the patient. The knowledge will be useless and the confidence and skill be wasted if at the same time the elementary details of practical nursing have not been so taught as to become an integral part of the nurse's professional being. The keynote of training should be the due co-ordination of theory and practice, so that on the one hand every item of theoretical knowledge can be exemplified in, or at least correlated with, some corresponding practical demonstration; and, on the other hand, for every detail of treatment an adequate reason can be given.

